# Dengue Viruses Are Enhanced by Distinct Populations of Serotype Cross-Reactive Antibodies in Human Immune Sera

**DOI:** 10.1371/journal.ppat.1004386

**Published:** 2014-10-02

**Authors:** Ruklanthi de Alwis, Katherine L. Williams, Michael A. Schmid, Chih-Yun Lai, Bhumi Patel, Scott A. Smith, James E. Crowe, Wei-Kung Wang, Eva Harris, Aravinda M. de Silva

**Affiliations:** 1 Department of Microbiology and Immunology, School of Medicine, University of North Carolina, Chapel Hill, Chapel Hill, North Carolina, United States of America; 2 Division of Infectious Diseases and Vaccinology, School of Public Health, University of California, Berkeley, Berkeley, California, United States of America; 3 Department of Tropical Medicine, Medical Microbiology and Pharmacology, University of Hawaii at Manoa, Honolulu, Hawaii, United States of America; 4 Departments of Medicine, Pediatrics, Pathology, Microbiology and Immunology, and The Vanderbilt Vaccine Center, Vanderbilt University Medical Center, Vanderbilt University, Nashville, Tennessee, United States of America; Harvard Medical School, United States of America

## Abstract

Dengue viruses (DENV) are mosquito-borne flaviviruses of global importance. DENV exist as four serotypes, DENV1-DENV4. Following a primary infection, individuals produce DENV-specific antibodies that bind only to the serotype of infection and other antibodies that cross-react with two or more serotypes. People exposed to a secondary DENV infection with another serotype are at greater risk of developing more severe forms of dengue disease. The increased risk of severe dengue in people experiencing repeat DENV infections appear to be due, at least in part, to the ability of pre-existing serotype cross-reactive antibodies to form virus-antibody complexes that can productively infect Fcγ receptor-bearing target cells. While the theory of antibody-dependent enhancement (ADE) is supported by several human and small animal model studies, the specific viral antigens and epitopes recognized by enhancing human antibodies after natural infections have not been fully defined. We used antibody-depletion techniques to remove DENV-specific antibody sub-populations from primary DENV-immune human sera. The effects of removing specific antibody populations on ADE were tested both *in vitro* using K562 cells and *in vivo* using the AG129 mouse model. Removal of serotype cross-reactive antibodies ablated enhancement of heterotypic virus infection *in vitro* and antibody-enhanced mortality *in vivo*. Further depletion studies using recombinant viral antigens showed that although the removal of DENV E-specific antibodies using recombinant E (rE) protein resulted in a partial reduction in DENV enhancement, there was a significant residual enhancement remaining. Competition ADE studies using prM-specific Fab fragments in human immune sera showed that both rE-specific and prM-specific antibodies in primary DENV-immune sera significantly contribute to enhancement of heterotypic DENV infection *in vitro*. Identification of the targets of DENV-enhancing antibodies should contribute to the development of safe, non-enhancing vaccines against dengue.

## Author Summary

The mosquito-borne dengue viruses (DENV) are responsible for approximately 390 million new infections worldwide each year, and an estimated 100 million of these infections lead to clinical disease. The presence of four different serotypes of DENV allows the same individual to experience more than one DENV infection. Secondary DENV infections with a different serotype are more likely to cause severe dengue disease than primary infections. One of the explanations for the greater risk of severe disease during secondary DENV infections is the phenomenon called antibody-dependent enhancement (ADE), where pre-existing DENV-specific antibodies enable entry of DENV into target host cells, and thereby enhance infection and disease. At the moment, the epitopes targeted by enhancing antibodies following a DENV infection are unclear. In the present study, we use novel techniques to fractionate human serum antibodies and test their ability to enhance DENV infection both *in vitro* (K562 cells) and *in vivo* (in a mouse model of lethal antibody-enhanced dengue disease). We found that antibodies binding both the envelope and prM proteins on the DENV virion play an important role in ADE of DENV by human immune sera. Our findings about DENV-enhancing antibodies in human immune sera are relevant to developing safe vaccines.

## Introduction

Dengue is present in over 100 countries and is the most common arthropod-borne viral disease of humans [1], [2]. Dengue disease is caused by dengue virus (DENV), which exists as four closely-related serotypes (DENV1-DENV4). DENV spreads between humans through the mosquito vectors *Aedes aegypti* and *Aedes albopictus*. Recent studies estimate that approximately 390 million individuals are infected with DENV globally each year, causing around 100 million clinically apparent cases [3]. There are currently no approved therapeutics or vaccines against DENV.

Primary DENV infections in humans result in type-specific as well as serotype cross-reactive antibodies. However, life-long protective immunity is only directed against the serotype of infection. During a secondary infection with another DENV serotype, individuals are at a greater risk of severe disease than during a primary infection [4]–[6]. Furthermore, in DENV-endemic regions, infants between the ages of 6 and 12 months are also a high-risk group for severe forms of dengue disease [7]–[9]. One of the most compelling explanations for the higher proportions of severe disease in infants and secondary heterotypic DENV infections is the phenomenon of antibody-dependent enhancement (ADE) [4]–[6]. ADE of DENV infection is expected to occur when pre-existing sub-neutralizing antibodies (e.g., from a primary infection) bind to a heterotypic virus during a subsequent infection and facilitate the entry of the virus through Fcγ receptor (FcγR)-mediated endocytosis into myeloid cells (such as monocytes and macrophages). Through mechanisms that are largely unclear, the antibody-bound virus escapes the phagolysosome and establishes a productive infection within the host cell [10]. Furthermore, productive DENV infections through ADE (as compared to the conventional route of entry) have been found to result in higher viremia and a suppressed host antiviral state [11]–[19].

Development of a suitable small animal model for the investigation of DENV infection and antibody responses has been hindered by the low or lack of DENV replication in immunocompetent mouse models. The first mouse models consisted of intracranial DENV challenges in immunocompetent suckling mice. However, these models resulted in death through neurological disease and paralysis, which are rarely seen in human dengue [20], [21]. DENV replication in a rodent model was first shown in the IFN-α/β and -γ receptor-deficient AG129 mouse model [22]. It was further demonstrated that the AG129 mouse model also presents a lethal vascular leakage syndrome with features similar to human disease when challenged with a high dose of DENV or a sub-lethal dose of DENV in the presence of DENV-specific enhancing antibodies [23]–[25]. Therefore, AG129 mice presently constitute the most suitable animal model available for testing antibodies for enhancement of DENV infections.

Recent studies investigating the memory B cell response after natural DENV infections have revealed that the antibody response in humans is dominated by cross-reactive, weakly neutralizing antibodies [26]–[29]. These cross-reactive antibodies were found to efficiently enhance DENV infection, usually over a wide range of concentrations [26]–[29]. A study analyzing the memory B cell response in humans after immunization with a monovalent formulation of a leading DENV vaccine candidate observed a similar dominantly cross-reactive, weakly neutralizing antibody response [30].

The specific antigens and epitopes on the virion targeted by enhancing antibodies in human immune sera have not been well defined. Nearly all DENV-specific antibodies, regardless of neutralization potency, will at some concentration enhance infection in FcγR-bearing cells. Thus, to investigate the viral epitopes targeted by antibodies responsible for enhancement of secondary DENV infection, it is insufficient to analyze enhancement properties of isolated human monoclonal antibodies (MAbs). Rather, the antibody repertoire in circulation prior to secondary infection needs to be examined, and ADE assays must be performed at antibody concentrations that approximate physiological concentrations in circulation or at the very least at low dilutions. The present study investigates the properties of antibodies in people exposed to primary infections that are responsible for enhancement of heterotypic DENV serotypes. The studies are conducted both *in vitro* (using the FcγR-bearing cell line, K562) and *in vivo* (using the AG129 mouse model). We demonstrate that primary DENV-immune human sera have distinct populations of antibodies that are responsible for DENV neutralization and ADE. The enhancing antibodies bind to serotype cross-reactive epitopes on envelope (E) and prM antigens on the viral surface.

## Results

People exposed to primary DENV infections develop a dominant serotype cross-reactive antibody response and a minor population of antibodies that are specific to the serotype of infection [31]. Previously, we demonstrated that the type-specific, and not the cross-reactive, antibodies were responsible for the ability of late convalescent primary DENV-immune sera to neutralize DENV [31]. Here we began by performing experiments to determine whether the dominant cross-reactive antibody response was responsible for ADE of DENV in both K562 cells and the AG129 mouse model.

**Figure 1:**
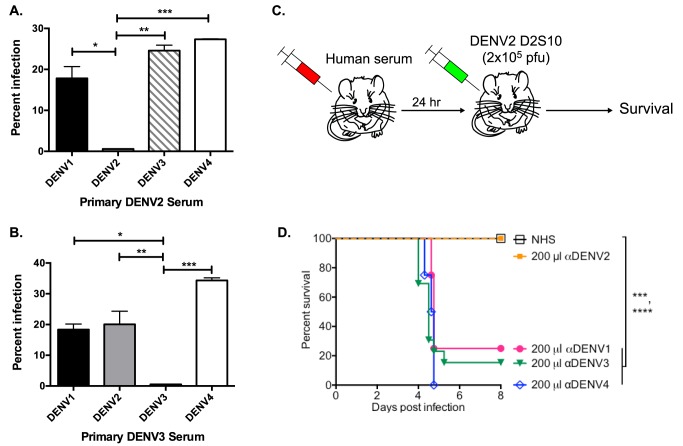
Primary DENV-immune human sera enhance heterotypic but not homotypic serotypes at high serum concentrations *in vitro* and *in vivo*. (**A and B**) Enhancement of heterotypic and homotypic DENV by primary DENV2-immune (DT 001) (**A**) and DENV3-immune (DT118) (**B**) sera at high serum concentration (1∶40) in K562 cells. Data are representative of 8 serum samples. **C**) Human serum was passively transferred to AG129 mice 24 hours prior to administration of 2×10^5^ pfu of the DENV2 strain D2S10 and survival of the mice was assessed. **D**) ADE-induced mortality in AG129 mice after D2S10 infection in the presence of heterotypic sera (primary DENV1, primary DENV3, and primary DENV4 sera) and homotypic human serum (primary DENV2 serum). Statistics: in Figure 1A, ADE of DENV2 compared to DENV1, *p = 0.01; DENV3, **p<0.001; and DENV4, ***p<0.0001; in Figure 1B, ADE of DENV3 compared to DENV1, *p = 0.02; DENV2, **p<0.005; and DENV4, ***p = 0.0003; in Figure 1D, NHS compared to anti-DENV1 serum, ***p = 0.0007; anti-DENV3 serum, ****p<0.0001; anti-DENV4 serum, ****p<0.0001.

We used the human erythromyeloblastoid leukemia cell line, K562, for investigation of enhancing antibodies in DENV-immune human sera. These cells, which express FcγRIIa, are only permissive to DENV infection in the presence of enhancing antibodies. At high serum concentrations (i.e., 1∶40 in **Figure 1A and B**), both primary DENV2- and DENV3-immune human sera enhanced heterotypic serotypes, but not the respective homotypic serotypes *in vitro*. Similarly, DENV-immune human sera were tested in the AG129 mouse model of antibody-enhanced dengue disease. The AG129 mice were passively administered human DENV-immune sera 24 hours prior to infection with the DENV2 strain D2S10 (**Figure 1C**). The homotypic immune human sera (i.e., primary DENV2-immune) did not induce any illness (data not shown) or death (**Figure 1D**) in mice challenged with a sublethal dose of DENV2, whereas passive transfer of the same quantity of heterotypic human serum (i.e., primary DENV1, DENV3 or DENV4-immune sera) led to significant ADE-induced disease (data not shown) and mortality (DENV-naïve human serum (NHS) compared to αDENV1, p = 0.0007; αDENV3, p<0.0001; αDENV4, p<0.0001).

### Depletion of Cross-Reactive Antibodies Ablates ADE of Heterotypic DENV

**Figure 2:**
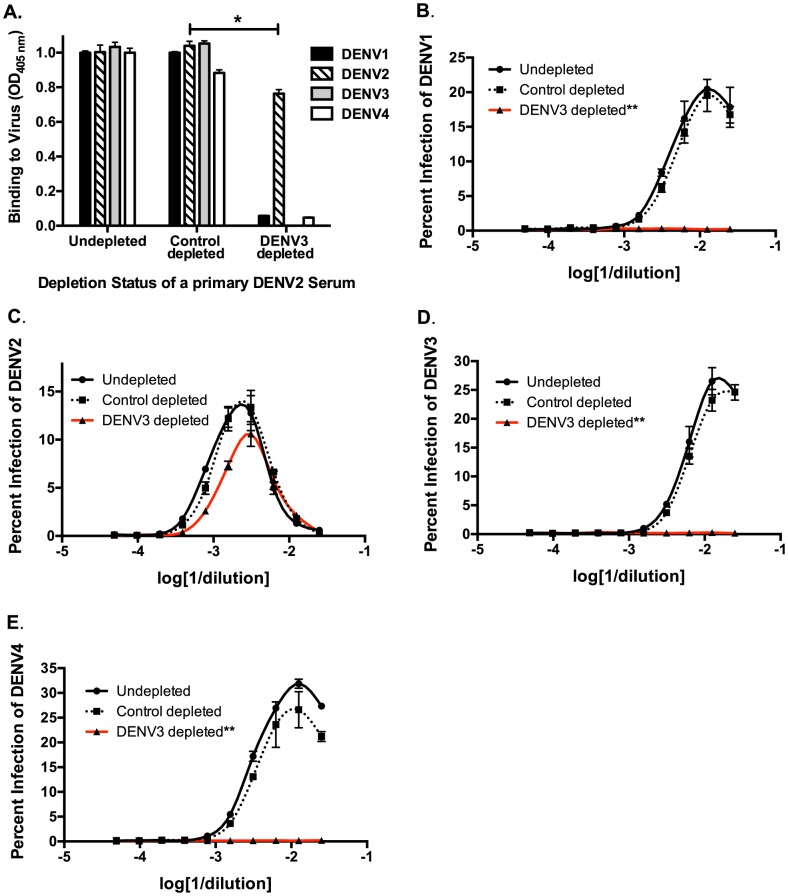
Removal of cross-reactive antibodies from primary DENV2-immune sera, eliminates enhancement of heterotypic DENV infection *in vitro*. **A**) DENV-binding ELISA shows that depletion of a primary DENV2-immune serum with DENV3 virus removes binding to heterotypic DENV1, DENV3 and DENV4. (**D–E**) In ADE assays performed in K562 cells, depletion of cross-reactive antibodies from primary DENV2-immune serum eliminated enhancement of heterotypic DENV1 (**B**), DENV3 (**D**), and DENV4 (**E**) but not homotypic DENV2 virus (**C**). Data are representative of at least four different primary DENV2-immune human sera. *Significantly different by Students t-test with p<0.01, **DENV3-depleted ADE curves significantly different from control-depleted ADE curves at serum dilutions 1∶40, 1∶80, 1∶160 and 1∶320 (Students t-test with p<0.001).

**Figure 3:**
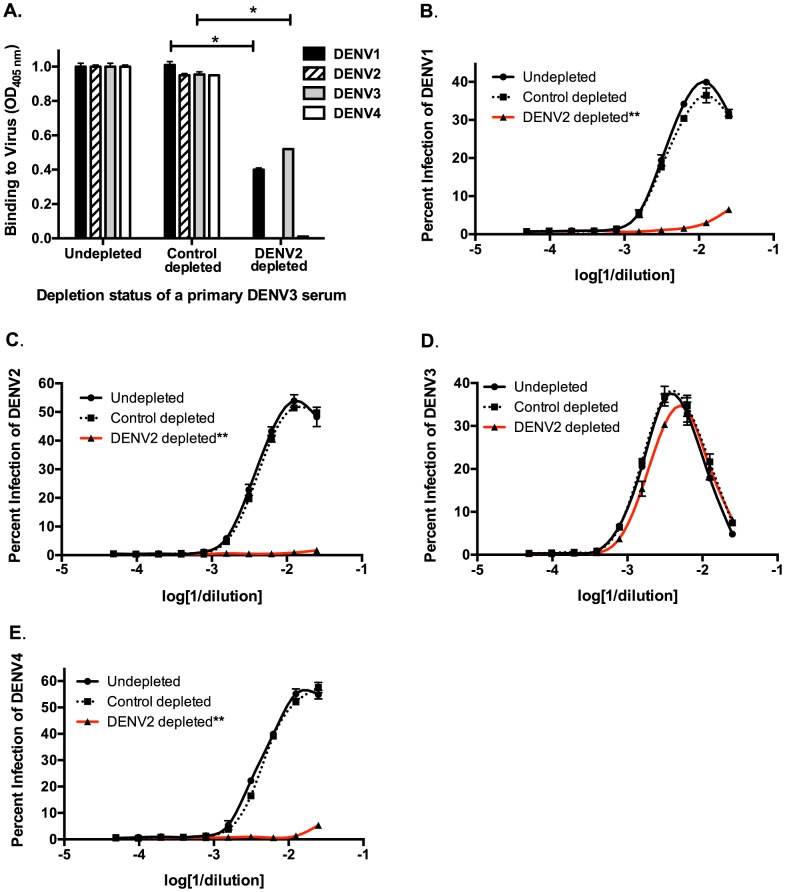
Removal of cross-reactive antibodies from primary DENV3-immune human sera abolishes enhancement of heterotypic DENV infection *in vitro*. **A**) DENV-binding ELISA shows that depletion of a primary DENV3-immune serum (DT118) with DENV2 virus removes binding to heterotypic DENV2 and DENV4, while significantly reducing binding to heterotypic DENV1. (**B–E**) In ADE assays performed in K562 cells, depletion of cross-reactive antibodies from primary DENV3-immune serum ablated enhancement of heterotypic DENV1 (**B**), DENV2 (**C**), and DENV4 (**E**), without reducing enhancement of homotypic DENV3 virus (**D**). Data are representative of at least four different primary DENV3-immune human sera. *Significantly different by Students t-test with p<0.01, **DENV2-depleted ADE curves significantly different from control-depleted ADE curves at serum dilutions 1∶40, 1∶80, 1∶160 and 1∶320 (Students t-test with p<0.001).

Next, we used *in vitro* and *in vivo* models to identify specific antibody populations in polyclonal sera that drive ADE. Primary DENV2-immune sera were depleted with the heterotypic virus DENV3, and primary DENV3-immune human sera were depleted with the heterotypic virus DENV2. As shown in **Figure 2A** and **Figure 3A**, successful virus-specific depletion was confirmed using a virus-binding ELISA. When primary DENV2-immune serum was depleted with DENV3 (**Figure 2A**), all cross-reactive binding antibodies were removed, and when primary DENV3-immune serum was depleted with DENV2, the remaining antibody bound to DENV3 and to a lesser extent to DENV1 (**Figure 3A**). This latter observation is consistent with known antigenic relationships between DENV serotypes; DENV1 and DENV3 share common epitopes that are not present in DENV2 or DENV4. *In vitro* ADE studies with heterotypic-virus depleted sera showed that removal of DENV3 virus-binding antibodies from primary DENV2-immune human sera completely ablated enhancement of the heterotypic serotypes, DENV1, DENV3 and DENV4 (**Figure 2B, D and E**), demonstrating that cross-reactive antibodies are responsible for enhancement of heterotypic serotypes. However, peak enhancement of the homotypic serotype, DENV2, was not affected by the removal of cross-reactive antibodies from DENV2-immune sera (**Figure 2C**), which suggests that homotypic enhancement only occurs when type-specific antibodies are diluted to low concentrations. Similar results were observed for primary DENV3-immune sera, where depletion of DENV2-specific antibodies completely removed all enhancement of infection by the heterotypic serotypes, DENV1, DENV2 and DENV4 (**Figure 3B, C, and E**), but not by the homotypic serotype DENV3 when diluted to low concentrations (**Figure 3D**).

**Figure 4:**
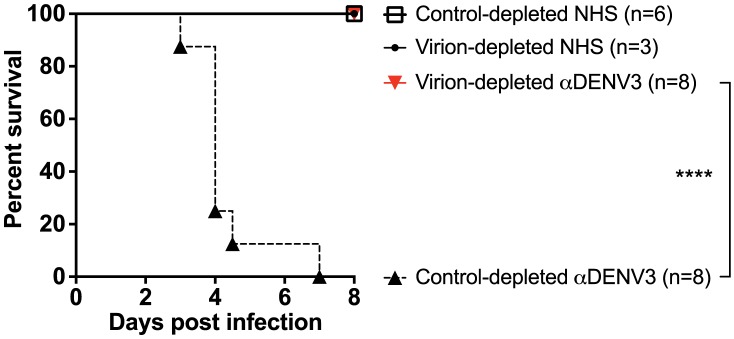
Removal of cross-reactive antibodies from primary DENV3-immune sera significantly protects AG129 mice from ADE-induced mortality. Primary DENV3-immune human sera (DT118 and DT105) were depleted with heterotypic DENV2 virus (resulting in depletion of heterotypic anti-DENV1, DENV2 and DENV4 antibodies), transferred to AG129 mice 24 hours prior to administration of 2×10^5^ pfu DENV2 (D2S10), and assessed for percent survival. ****Percent survival of virion-depleted DENV3 serum is significantly different from control-depleted DENV3 serum (p<0.0001). Data are pooled from three independent experiments using two primary DENV3-immune human sera.

DENV-immune sera depleted of all cross-reactive antibodies were then transferred into AG129 mice to assess the role of cross-reactive antibodies in DENV enhancement *in vivo*. Passive administration of control-depleted, primary DENV3-immune sera into AG129 mice prior to infection with a sublethal dose of DENV2 D2S10 resulted in 100% mortality via ADE (**Figure 4**). However, removal of heterotypic virus-binding antibodies from primary DENV3-immune sera (by depleting with DENV2) led to 100% survival (p<0.0001 comparing groups administered DENV2 virion- or control-depleted sera), similar to mice administered virion- or control-depleted DENV-naïve human sera (**Figure 4**). Thus, cross-reactive antibodies binding to the virion (i.e., the structural proteins) are the main antibody component in human immune serum responsible for heterotypic DENV enhancement both in cell culture and in the ADE mouse model.

### Depletion of Recombinant E-Specific Antibodies Decreases ADE of Heterotypic DENV

**Figure 5:**
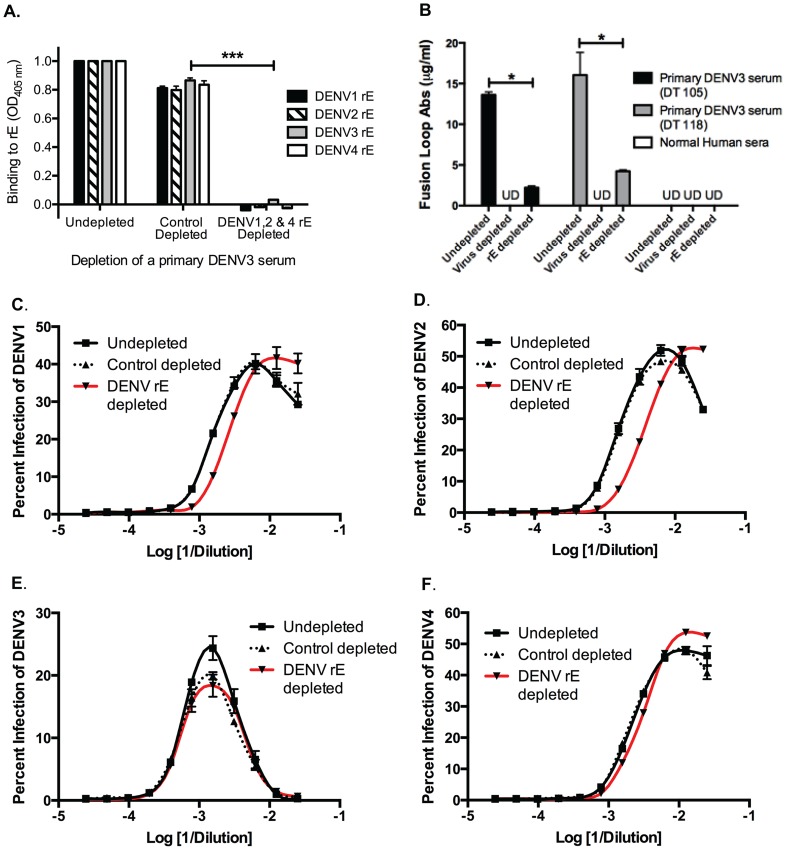
DENV E-binding antibodies partially contribute to heterotypic virus-enhancing antibodies *in vitro*. **A**) Depleting primary DENV3 serum (DT118) with rE of DENV1, DENV2 and DENV4 removed rE-binding antibodies as assessed by rE binding ELISA. **B**) The quantity of fusion loop-specific antibodies in virus-depleted and rE-protein depleted sera was assessed using a virus-binding assay with wild-type and fusion loop-mutated virus-like particles (*significantly different by Students t-test with p<0.02, ***p<0.001). UD: undetectable due to antibody concentration being below the limit of detection. (**C–F**) In ADE assays performed with human K562 cells, removal of cross-reactive rE-specific antibodies from a primary DENV3-immune human serum partially reduced enhancement of the heterotypic viruses DENV1 (**B**) and DENV2 (**C**), but not DENV4 (**E**) or the homotypic virus, DENV3 (**D**). In panels C and D the DENV1-, 2-, and 4-depleted ADE curves were significantly different from control-depleted ADE curves at serum dilutions 1∶320, 1∶640, and 1∶1280 (Students t-test with p<0.05). Data are representative of four DENV-immune sera.

We next investigated the role of DENV E protein-binding antibodies in heterotypic DENV enhancement. Primary DENV-immune human sera were depleted of cross-reactive E-binding antibodies using heterotypic purified recombinant DENV E protein (rE). Removal of cross-reactive rE-binding antibodies from primary DENV3-immune serum eliminated binding to the homotypic DENV3 rE protein as well, indicating that a majority of the antibodies binding this rE protein construct in DENV-immune sera were cross-reactive (**Figure 5A**). The fusion loop region in E protein domain II is a target of DENV cross-reactive human antibodies. Using dengue virus-like-particles (VLP) with mutations in the E protein fusion loop epitopes, we confirmed that most fusion loop antibody was removed following rE depletion (**Figure 5B**). *In vitro* cell culture-based ADE investigations showed that removal of cross-reactive DENV rE-binding antibodies from DENV3-immune sera led to a partial decrease (i.e., a shift of the curve to the right) in the enhancement potency of the immune sera against heterotypic serotypes DENV1 and DENV2, but not DENV4 (**Figure 5C–F**). Similar observations were seen with rE-depleted DENV2-immune human sera against the heterotypic DENV serotypes (data not shown). These results indicate that rE-binding cross-reactive antibodies are at least partially responsible for heterotypic enhancement.

**Figure 6:**
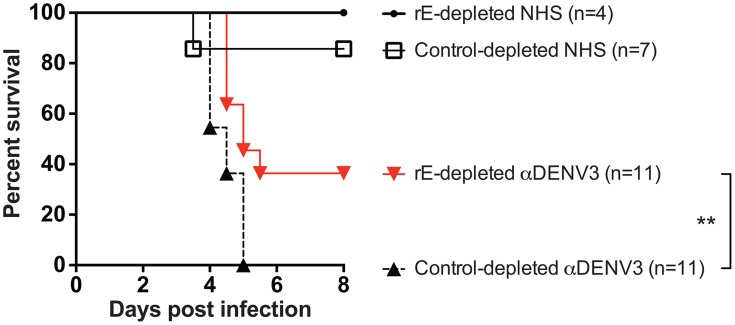
DENV E-specific antibodies in human serum are *partially* responsible for ADE of heterotypic DENV infections in AG129 mice. **A**) Two primary DENV3-immune sera were depleted of cross-reactive DENV rE-specific antibodies, transferred to AG129 mice 24 hours prior to administration of 2×10^5^ pfu of DENV2 D2S10 virus, and assessed for percent survival. Data are pooled from 5 independent experiments using two different primary DENV3-immune human sera (i.e. DT118 and DT105). **rE-depleted DENV3-immune serum data were significantly different from the control-depleted DENV3-immune serum data (p = 0.0098).

The DENV enhancement properties of sera depleted of cross-reactive, rE-binding antibodies were tested *in vivo*. Removal of cross-reactive rE-binding antibodies resulted in survival of 40% of the mice, which was significantly (p = 0.0098) different from mice receiving the control-depleted sera where none of the animals survived (**Figure 6**). However, the 40% survival following depletion of cross-reactive rE-binding antibodies was also significantly (p = 0.0062) different from mice receiving the virion-depleted α-DENV3 sera, where all the animals survived (**Figures 4** **and** **6**). Similar results were observed for two different primary DENV3-immune sera. Thus, DENV rE-binding cross-reactive antibodies in primary DENV-immune human sera were partially responsible for ADE of heterotypic DENV infections *in vivo* as well.

### Competition ADE Assays of Human DENV-Immune Sera with prM-Binding Fab Fragments Indicate a Role for prM-Binding Antibodies in ADE

**Figure 7:**
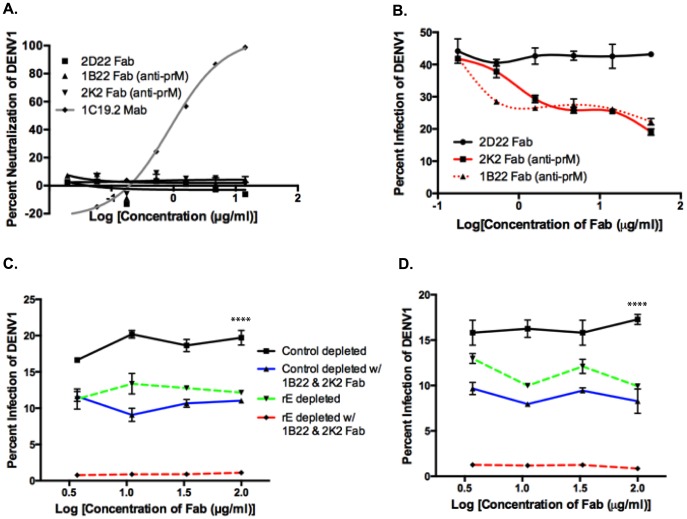
prM-specific and rE-specific cross-reactive antibodies together make up the entire population of enhancing antibodies in DENV-immune human sera. **A**) DENV1 neutralization by Fab 1B22 (anti-prM), Fab 2K2 (anti-prM), Fab 2D22 (DENV2-specific, negative control), and MAb 1C19.2 (DENV1-neutralizing, positive control) in U937-DC-SIGN cells. **B**) Competition ADE assay in K562 cells of Fab fragments with primary DENV3-immune serum (at peak enhancement concentration) using DENV1. Competition ADE assay of Fab fragments with rE-specific antibody-depleted DENV3-immune sera, DT118 (**C**) and DT105 (**D**) using DENV1 (at peak enhancement concentration) in K562 cells. ****Control depleted curves significantly different from control-depleted with 1B22 & 2K2 Fab (p<0.0001), rE-depleted (p<0.0001), and rE-depleted with 1B22 & 2K2 Fab (p<0.0001) by nonparametric ANOVA test.

Since we found that rE-binding antibodies were only partially responsible for enhancement of heterotypic serotypes, we investigated the role of prM-binding antibodies in human immune sera. prM is a small integral membrane protein that is difficult to express and purify as a recombinant antigen. Thus, we probed the importance of prM-binding antibodies by conducting competitive ADE assays with primary DENV-immune sera and Fab fragments from human MAbs that bound to prM. The competition ADE assay was developed on the basis that at high concentrations, prM Fab fragments, which cannot bind Fc receptors since they lack the Fc portion of the antibody, should bind to DENV and prevent the binding of potentially enhancing intact prM antibodies in serum. Fab fragments were generated from the prM-binding MAbs 1B22 and 2K2 by proteolytic cleavage. Both MAbs 1B22 and 2K2 were isolated from memory B cells following secondary DENV infections (**Table S1**), and were mapped to prM by Western blot and prM-binding ELISA (data not shown). As shown in **Figure 7A** and **Table S1**, neither Fab 1B22 nor Fab 2K2 neutralized DENV1 at the concentrations used in the competitive ADE assay. Enhancement of the heterotypic serotype, DENV1, by primary DENV3-immune sera was not affected by the increasing presence of a negative control binding Fab, 2D22 Fab (DENV2-specific) (**Figure 7B**). However, addition of Fab 1B22 or Fab 2K2 competed for virus binding with DENV-specific antibodies in the DENV3-immune serum and reduced enhancement of heterotypic virus infection by 25–50% in a dose-dependent manner (**Figure 7B**). Thus, it appears that prM-binding antibodies in primary immune serum also play a role in ADE of heterotypic DENV serotypes.

### Both E- and prM-Specific Antibodies Together Account for a Majority of the Heterotypic ADE Observed *In Vitro*

We then utilized control-depleted and rE-antibody depleted DENV-immune sera in the competition ADE assay to assess the cumulative effect of both rE- and prM-specific antibodies to enhancement of heterotypic DENV serotypes. As depicted in **Figure 7C and D**, addition of prM Fab fragments to rE-depleted primary DENV3-immune sera led to a complete loss of infection enhancement of the heterotypic serotype, DENV1. As prM lies in close proximity to the fusion loop on E protein, it is conceivable that prM-binding Fab fragments not only block the binding of other prM-specific antibodies, but also interfere with fusion loop-binding antibodies. To test this possibility, we performed competition-binding assays with prM Fab fragments and full-length MAbs against fusion loop epitopes (**Figure S1A** and **B**), EDIII epitopes (**Figure S1C**) and prM epitopes (**Figure S1D**). While we observed strong competition between the anti-prM Fab fragments and the full-length anti-prM MAb 2H2 (**Figure S1D**), no competition was observed between anti-prM Fab fragments and fusion loop-binding (**Figure S1A** and **B**) or EDIII-directed MAbs (**Figure S1C**). Our results thus indicate that both fusion loop and prM antibodies in human DENV-immune sera independently contribute to ADE.

## Discussion

On average, people exposed to secondary DENV infections have a higher viremia and an increased risk of developing dengue hemorrhagic fever/dengue shock syndrome (DHF/DSS) compared to people experiencing primary infections [1], [5]. ADE has been proposed as an explanation for the increased risk of DHF/DSS in infants born to DENV-immune mothers or in people exposed to secondary DENV infections. The enhancement of DENV infection by antibodies has been directly demonstrated in cell culture and animal models of infection [12], [17], [23], [25]. The specific properties of antibodies in primary DENV-immune human sera that are likely to enhance an infection with a new serotype have not been definitively identified. Attempts to study specific properties of enhancing antibodies has been complicated by the observation that almost any DENV-specific antibody, including strongly neutralizing antibodies, can enhance infection at sub-neutralizing concentrations. The goal of the current study was to use human immune sera at concentrations likely to exist in people susceptible to secondary DENV infections and to define the properties of enhancing antibodies in this polyclonal antibody mixture.

Our results reported here, together with previous studies on neutralizing antibodies, demonstrate that primary DENV-immune sera consist of one population of type-specific antibodies that neutralize the serotype (homotypic) of previous infection [31], and a second population of serotype cross-reactive antibodies that enhance new (heterotypic) serotypes. The envelope of DENV contains E and prM proteins, and cross-reactive epitopes on both these antigens are targeted by enhancing antibodies in human sera. Our results indicate that prM- and E-specific antibodies each contributed separately to ADE. Numerous DENV cross-reactive, weakly neutralizing and strongly enhancing MAbs isolated from both mice and human dengue cases have been mapped to the highly conserved flavivirus fusion loop/peptide region at the tip of domain II of E protein [32]–[35]. Our data support the idea that the fusion loop is an important target of DENV-enhancing antibodies.

In addition to E-specific antibodies, several groups have recently isolated many weakly neutralizing, cross-reactive prM-binding antibodies from human PBMCs from individuals after natural primary DENV infections [26]–[30]. Similar to MAbs 1B22 and 2K2 used in the present study, a majority of the isolated human prM-specific MAbs have been mapped to the soluble, pr portion of the prM protein [29], [30], [33], [36], [37]. This suggests that the soluble pr portion may be the major target of human antibodies on the prM protein. The X-ray crystallographic structure of the prM-E heterodimer shows that the pr portion of prM sits directly above the tip of EDII and partially covers the fusion loop of the E protein, protecting it from premature fusion within the Golgi apparatus [38]. In addition, detailed mapping analysis of these prM antibodies has revealed epitopes on the pr protein that are in close proximity to the fusion loop [37], [39]. Furthermore, a recent study showed that non-FcγR binding variants of fusion loop-specific MAbs can be used to therapeutically prevent enhancement by anti-DENV-immune mouse and human sera which contain a mixture of E- and prM-specific antibodies [40]. Therefore, it was conceivable that our competition ADE assay with prM-binding Fab fragments not only blocked the binding of prM-specific antibodies, but also interfered with fusion loop-binding antibodies. However, we did not observe significant competition between the prM Fab fragments and whole MAbs binding to fusion loop and other E protein epitopes. We conclude that both fusion loop- and prM-binding antibodies in immune sera may independently contribute to ADE.

The prM protein is proteolytically cleaved by the host enzyme, furin, during maturation of DENV in the Golgi apparatus. Therefore, virus binding and enhancement of infection by prM-specific antibodies is restricted to the prM-containing virion particles that are either immature or partially immature. The cell culture production of DENV yields viral particles with a range of maturity, consisting of fully mature, partially mature and fully immature virions in the same virus mix [41]. Published work has shown that prM-antibodies can even facilitate entry and productive infection of fully immature virion particles [10]. However, the maturity of DENV particles produced in humans during a natural infection is unknown. Therefore, the role of prM-binding antibodies in enhancement or neutralization during a natural infection is still unclear.

Viral epitopes recognized by enhancing and neutralizing antibodies appear to be different both in location and complexity. Several studies have identified the hinge region between domains I and II of the E protein as the target of type-specific neutralizing antibodies in human immune sera [31], [42]–[44]. The neutralizing antibodies bind to quaternary epitopes that only form after virus assembly. In contrast, the enhancing epitopes appear to be simpler in structure because they are preserved on soluble, recombinant forms of E and prM proteins. Nevertheless, additional considerations such as virus maturation state and the differential surface exposure of epitopes in virus versus recombinant proteins need to be considered when extrapolating from these model systems to human infections.

Three main human FcγRs (FcγRI, FcγRIIa and FcγRIIb) have been shown to play roles in ADE of DENV in host cells [45]–[47]. Although both human FcγRI and FcγRIIa are activating Fcγ receptors, only FcγRIIa contains an internal ITAM motif. The low-affinity FcγRIIb is an inhibitory receptor due to the presence of an ITIM motif [48]. Both FcγRI and FcγRIIa facilitate the uptake of IgG-bound virus immune complexes into the host cell, while cross-linking of FcγRIIb with DENV-bound IgG inhibits uptake of these immune complexes at high antibody concentrations [47]. K562 cells contain the low affinity FcγRIIa, but lack both the high affinity FcγRI and the inhibitory FcγRIIb [45]. However, unlike FcγRI, FcγRIIa is more widely expressed in immune cells, binds all four subclasses of IgG, is more efficient at the uptake of IgG-bound DENV immune complexes, and has a polymorphism in humans that is correlated with greater risk for severe dengue disease [46], [49]–[51]. Therefore, although the absence of FcγRIIb in K562 cells is a valid concern, the absence of FcγRI may not significantly affect the observed data.

The present study demonstrates that a subpopulation of DENV-specific antibodies in human immune sera were responsible for ADE in cell culture and in a mouse model of severe dengue disease. These studies further establish the AG129 mice as a good model to study ADE. AG129 mice contain the full repertoire of mouse Fcγ receptors, which are capable of binding human IgG. Our demonstration with model systems *in vitro* and *in vivo* that DENV serotype cross-reactive antibodies in human immune sera are responsible for enhanced replication and disease is consistent with the ADE hypothesis proposed for severe dengue in people.

## Methods

### Viruses and Cell Lines

All *in vitro* assays were conducted with the DENV WHO reference strains, i.e., DENV1 West Pac 74, DENV2 S-16803, DENV3 CH54389 and DENV4 TVP-360, which were initially obtained from Dr. Robert Putnak (Walter Reed Army Institute of Research, Silver Spring, MD). All *in vivo* assays in the AG129 mice were conducted using the mouse-adapted DENV2 D2S10 strain, which has two defined mutations in the E protein that result in reduced viral clearance [24], [52]. All viruses were grown as described previously using the *Aedes albopictus* mosquito cell line C6/36 [53]. All viruses for antigen purification were grown in the African green monkey kidney epithelial cell line, Vero. *In vitro* ADE and neutralization assays were performed using human erythromyeloblastoid leukemia K562 cells and DC-SIGN-expressing U937 cells (U937-DC-SIGN), respectively. K562 cell lines were obtained from ATCC, while U937-DC-SIGN were kindly provided by the laboratory of Dr. Mark Heise at the University of North Carolina, Chapel Hill.

### Human Sera and Fab Fragments

#### Ethics statement

Blood donations were obtained from individuals who had traveled to dengue-endemic regions and experienced a primary DENV1, -2, -3, or -4 infection. These human samples were obtained with informed consent approximately 2 to 10 years after DENV infection. All blood donations were collected in compliance with the Institutional Review Board of the University of North Carolina at Chapel Hill. Written informed consent was obtained from all subjects before participation in the study. All samples were coded and analyzed anonymously. At least four DENV2-immune sera and four DENV3-immune sera were tested. DT105 and DT118 are late convalescent human sera from past primary DENV3 infections, and have been described previously [31].

The human MAbs 1F4 (DENV1-specific, EDI-EDII hinge binding), 2D22 (DENV2-specific, EDI-DII hinge binding), 1C19.2 (cross-reactive, EDIII-binding), 1B22 (cross-reactive, prM-binding) and 2K2 (cross-reactive, prM-binding) were generated in collaboration with Dr. Scott Smith in the laboratory of Dr. James Crowe (Vanderbilt University) [30]. The Fab fragments of 1F4, 2D22, 1C19.2, 1B22 and 2K2 were generated through enzymatic papain digestion of the respective MAbs. Neutralization and binding properties of all human MAbs used in present study are displayed in **Table S1**.

### Depletion of Virus-Specific Antibodies from Human Sera

Human sera were depleted of virus-specific antibodies as previously described [31]. Briefly, DENV was grown in Vero cells and purified using ultracentrifugation, sucrose cushion and Opti-prep gradients as described previously [31], [53]. The highly purified DENV was then passively adsorbed to polystyrene beads (4.5 µm) and incubated with human sera at 37°C to remove the appropriate DENV-specific antibodies. Control depletion entailed incubation of serum with polystyrene beads coated with BSA. Successful depletion was assessed using a virus-binding ELISA.

### Depletion of rE-Specific Antibodies from Human Sera

Human sera were depleted of rE-binding antibodies as previously described [31]. Briefly, purified recombinant E proteins (soluble domain) from all four DENV serotypes were purchased from Hawaii Biotech, Inc. Purified rE was covalently conjugated to cyanogen bromide (CNBr)-activated beads using amine chemistry. We have previously demonstrated that the major antibody-binding epitopes are preserved on rE after covalent attachment to CNBr-activated beads [31]. The rE-conjugated beads were then incubated with human sera at 37°C to remove rE-specific antibodies. Control depletion consisted of depleting serum with CNBr-activated beads that were conjugated to BSA. Successful removal of all cross-reactive rE-specific antibodies was confirmed using a rE-binding ELISA.

### ELISA Binding Assays

Binding of depleted human sera to purified DENV or rE protein was measured using ELISA binding assays as previously described [31]. Briefly, DENV virions or rE proteins were either directly coated or captured by the anti-E protein mouse Mab 4G2, blocked with 1% normal goat serum (Gibco Life Technologies, USA), and incubated with human serum diluted 1∶20, and binding was detected with an alkaline phosphatase-conjugated anti-human secondary antibody.

The above protocol was also followed for *competition binding ELISA assays* with the difference that the Fab fragments, 1F4 or 1B22 and 2K2, were added to serially diluted (0.0005–8.0 µg/ml) mouse MAbs prior to incubation with purified DENV2 coated on ELISA plates. The mouse MAbs used were E protein fusion loop-binding (4G2 and MAb 30), EDIII-binding (12C1) or prM-binding (2H2). Fab fragments were used at 1.0 µg/ml for this competition assay.

### Quantification of Anti-fusion Loop Antibodies Using Wild-Type and Mutant VLPs

DENV fusion loop-binding antibodies in human polyclonal serum were assessed using wild-type (WT) DENV1 (produced by pCB-D1 construct) and fusion loop mutant (W101A and F108A) VLPs as described previously [35], [54]. Briefly, WT and mutant VLPs were captured in a 96-well plate coated with anti-DENV1 rabbit anti-serum. The wells were incubated with a two-fold dilution series of the human immune sera, followed by incubation with anti-human IgG conjugated to horseradish-peroxidase, then TMB substrate and finally stop solution. The percentage of fusion loop-binding antibodies was calculated using the formula: % anti-fusion loop antibodies = [1-endpoint titer to mutant VLPs/endpoint titer to WT VLPs]×100% [35], [54].

### U937-DC-SIGN Neutralization Assay

The flow cytometry-based DENV neutralization assay was conducted as described previously [31]. Briefly, human serum was serially diluted and incubated with DENV for 1 hour at 37°C under 5% CO_2_. Virus-antibody mixture was then added to U937-DC-SIGN cells for 2 hours at 37°C, after which cells were washed 2 times with fresh medium, and then incubated for 22 hours. Twenty-four hours post-infection, the cells were washed with fresh medium, fixed with 4% paraformaldehyde, and stained with 2H2 antibody conjugated to Alexa-488, and the percentage of infected cells was measured by flow cytometry.

### ADE Assay in K562 Cells

*In vitro* ADE assays were conducted in K562 cells as previously described [55]. Human sera were diluted 2-fold starting from 1∶20, then incubated for 1 hour at 37°C with DENV at an MOI of 1.0. Approximately 5×10^4^ cells were added to each well containing virus-antibody mixtures and then incubated for 2 hours at 37°C, after which cells were washed 2× with fresh medium and incubated at 37°C for another 22 hours. Cells were fixed 24 hours post-infection, stained for DENV E protein using MAb 2H2 (anti-prM) conjugated to Alexa-488 and analyzed by flow cytometry to measure percent infection. The *competition ADE assays* were conducted similarly, except for the difference that the Fab fragments were serially diluted and combined with DENV3-immune primary sera (at peak enhancing concentration) prior to incubation with DENV1 for 1 hour.

### ADE Assay in AG129 Mice

*In vivo* ADE assays in AG129 mice were conducted at UC Berkeley in the Animal Facility in accordance with Animal Care and Use Committee Guidelines as previously described [55]. AG129 mice were administered DENV-immune human sera or normal human serum (NHS) intraperitoneally in a final volume of 200 µl, approximately 24 hours prior to intravenous administration of a sub-lethal 2×10^5^ pfu dose of DENV2 D2S10. Mice were then observed over a 10-day period or until euthanized and scored for morbidity and mortality using a standardized 5-point system [56].

### Statistical Analysis

Statistical analysis of Kaplan-Meier survival blots was performed using the Log-rank (Mantel-Cox) test. Students t-test was used for the *in vitro* ELISA virus-binding, rE-binding experiments, and fusion loop antibody quantification experiments. The nonparametric ANOVA test was used for several ADE experiments.

## Supporting Information

Figure S1 **prM-specific Fab fragments compete with the binding of other prM-specific MAbs, but not E protein fusion loop or EDIII-binding MAbs.** Competition binding ELISA assays were conducted with purified DENV2 virus. The binding of fusion loop-specific mouse MAbs, 4G2 (**A**) and MAb 30 (**B**), EDIII-specific mouse MAb, 12C1 (**C**), and prM-binding mouse MAb, 2H2 (**D**), were competed using either no Fab, DENV1-specific Fab 1F4, or prM-specific Fabs, 1B22 and 2K2. MAbs were titrated down and Fabs were added for competition at a concentration of 1 µg/ml.
(DOCX)

Table S1 **DENV binding and neutralization profiles of human MAbs used in the present study.**
(DOCX)
