# Supplementary material for: Dengue Viruses Are Enhanced by Distinct Populations of Serotype Cross-Reactive Antibodies in Human Immune Sera
Source: PLoS Pathog. 2014 Oct 2;10(10):e1004386. doi: 10.1371/journal.ppat.1004386 (PMC4183589; doi:10.1371/journal.ppat.1004386)
Supplement: Figure S1 — prM-specific Fab fragments compete with the binding of other prM-specific MAbs, but not E protein fusion loop or EDIII-binding MAbs. Competition binding ELISA assays were conducted with purified DENV2 virus. The binding of fusion loop-specific mouse MAbs, 4G2 (A) and MAb 30 (B), EDIII-specific mouse MAb, 12C1 (C), and prM-binding mouse MAb, 2H2 (D), were competed using either no Fab, DENV1-specific Fab 1F4, or prM-specific Fabs, 1B22 and 2K2. MAbs were titrated down and Fabs were added for competition at a concentration of 1 µg/ml. (DOCX) [file ppat.1004386.s001.docx]

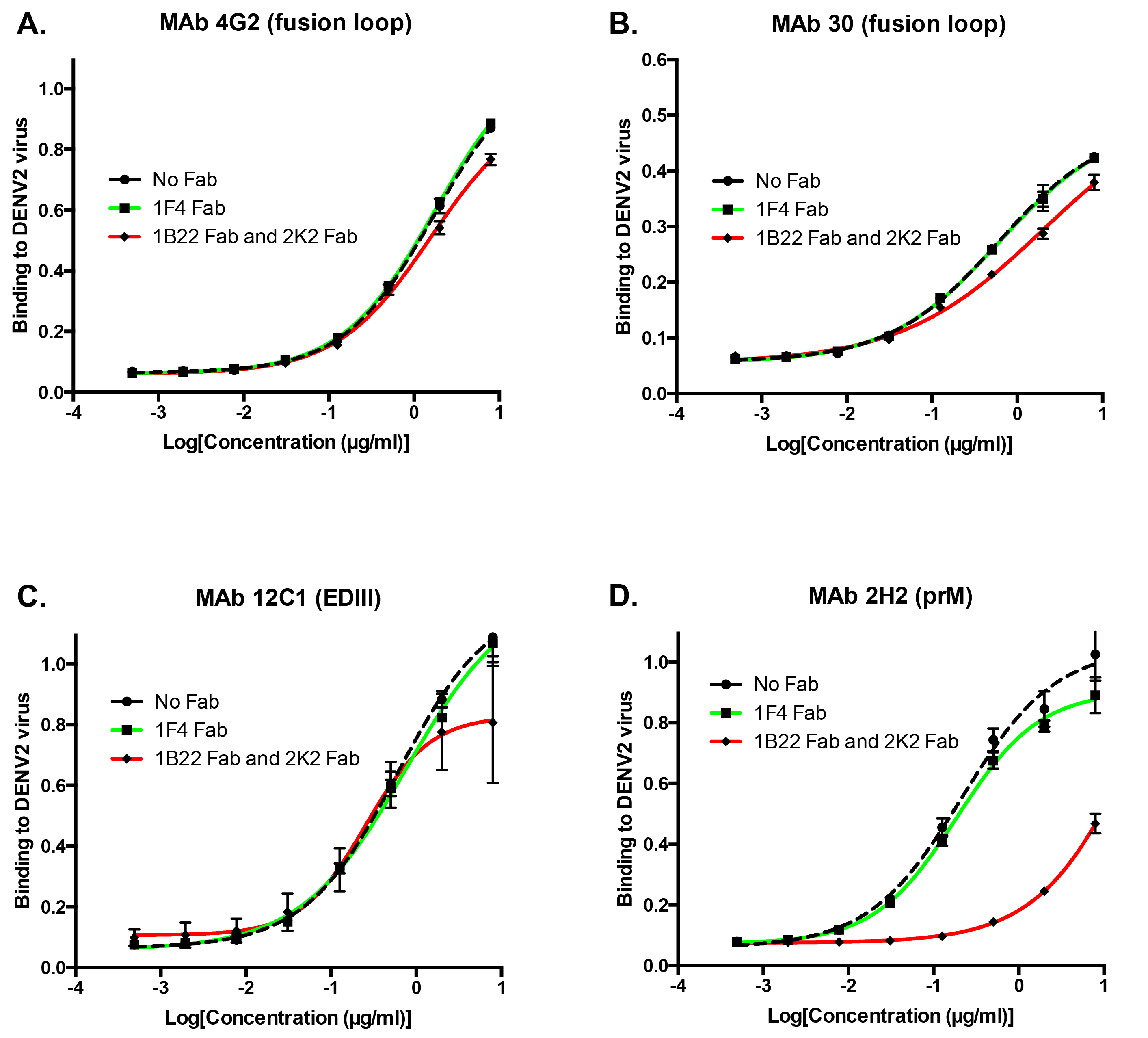


**Figure S1. prM-specific Fab fragments compete with the binding of other prM-specific MAbs, but not E protein fusion loop or EDIII-binding MAbs.** Competition binding ELISA assays were conducted with purified DENV2 virus. The binding of fusion loop-specific mouse MAbs, 4G2 (**A**) and MAb 30 (**B**), EDIII-specific mouse MAb, 12C1 (**C**), and prM-binding mouse MAb, 2H2 (**D**), were competed using either no Fab, DENV1-specific Fab 1F4, or prM-specific Fabs, 1B22 and 2K2. MAbs were titrated down and Fabs were added for competition at a concentration of 1 μg/ml.
