# Supplementary material for: Dengue Viruses Are Enhanced by Distinct Populations of Serotype Cross-Reactive Antibodies in Human Immune Sera
Source: PLoS Pathog. 2014 Oct 2;10(10):e1004386. doi: 10.1371/journal.ppat.1004386 (PMC4183589; doi:10.1371/journal.ppat.1004386)
Supplement: Table S1 — DENV binding and neutralization profiles of human MAbs used in the present study. (DOCX) [file ppat.1004386.s002.docx]

**Table S1. DENV binding and neutralization profiles of human MAbs used in the present study.**

| Human MAb | Donor | Binding (at 2 μg/ml) | | | | 50% Neutralization titer (μg/ml) | | | |
| --- | --- | --- | --- | --- | --- | --- | --- | --- | --- |
|  |  | Virus | E | EDIII | prM^2^ | DENV1 | DENV2 | DENV3 | DENV4 |
| 1B22 | Secondary | Cross-reactive | - | - | + | >10 | >10 | >10 | >10 |
| 1C19.2 | Secondary | Cross-reactive | + | + | - | 0.22 | >10 | >10 | >10 |
| 1F4^1^ | Secondary | Type-specific (DENV1) | - | - | - | 0.11 | >10 | >10 | >10 |
| 2D22^1^ | DENV2 Primary | Type-specific (DENV2) | - | - | - | >10 | 0.10 | >10 | >10 |
| 2K2 | Secondary | Cross-reactive | - | - | + | >10 | >10 | >10 | >10 |

^1^DENV Binding and neutralization data for the human Mabs 1F4 and 2D22 were taken from Smith, S. et al. 2012, and de Alwis, A.R. et al. 2012 [1,2].

1. de Alwis R, Smith SA, Olivarez NP, Messer WB, Huynh JP, et al. (2012) Identification of human neutralizing antibodies that bind to complex epitopes on dengue virions. Proc Natl Acad Sci U S A 109: 7439-7444.

2. Smith SA, Zhou Y, Olivarez NP, Broadwater AH, de Silva AM, et al. (2012) Persistence of circulating memory B cell clones with potential for dengue virus disease enhancement for decades following infection. J Virol 86: 2665-2675.
